# The Psychology of Laughing

**Published:** 1898-11

**Authors:** 


### The Psychology of Laughing.

Drs. G. S. Hall and A. Allen have analyzed the results, which are reported in the *Occidental Med. Times*, of an examination of nearly three thousand persons, and found that the first indication of laughter may in exceptional cases be seen in almost any part of the body, but it is most frequent in the eyes, and next in frequency at the mouth. The eyes become brighter, smaller and oscillate, and the mouth opens, stretches and curves upward, but sometimes downward. In a few cases the laugh begins with dimples in the cheeks, in others with a movement of the muscles just below the ears or a throwing back of the head. Subjectively, the "funny feeling" may begin in the stomach, throat, head, diaphragm, face, etc. Sometimes beauty is evoked or increased, or ugliness is produced. The eyes sometimes open, sometimes shut, sometimes grow dull, both lids may tremble and the eyeballs may twitch, grow rigidly fixed or roll wildly, or they may be turned upward and inward, and they are often suffused with tears. The after-effects of a hearty laugh were described as exhaustion, heavy breathing, fatigue, shame, weakness, soberness, sadness, relief, weakness localized in various parts of the body, deep sighs, giddiness, perspiration, headache, stitch in the side, soreness, thirst chills, sleepiness, uncontrollable movements, nausea, tears, fear of impending disaster, breathlessness, etc. A laugh is, according to Dr. Hall, not unlike an epilepsy from the aura, at which stage it may be checked, to the subsequent exhaustion. In a number of cases laughter was evoked by news or sights really sad.—*Maryland Med. Journal.*

